# Mechanisms of stress response in the root stem cell niche

**DOI:** 10.1093/jxb/erab274

**Published:** 2021-06-10

**Authors:** Elena V Ubogoeva, Elena V Zemlyanskaya, Jian Xu, Victoria Mironova

**Affiliations:** 1 Institute of Cytology and Genetics, Novosibirsk, Russia; 2 Novosibirsk State University, Novosibirsk, Russia; 3 Department of Plant Systems Physiology, Institute for Water and Wetland Research, Radboud University, Heyendaalseweg 135, 6525 AJ, Nijmegen, The Netherlands; 4 Universidad Nacional Autónoma de México, Mexico

**Keywords:** Auxin, DNA damage, quiescent center, regeneration, ROS, stem cell niche, stress

## Abstract

As plants are sessile organisms unable to escape from environmental hazards, they need to adapt for survival. The stem cell niche in the root apical meristem is particularly sensitive to DNA damage induced by environmental stresses such as chilling, flooding, wounding, UV, and irradiation. DNA damage has been proven to cause stem cell death, with stele stem cells being the most vulnerable. Stress also induces the division of quiescent center cells. Both reactions disturb the structure and activity of the root stem cell niche temporarily; however, this preserves root meristem integrity and function in the long term. Plants have evolved many mechanisms that ensure stem cell niche maintenance, recovery, and acclimation, allowing them to survive in a changing environment. Here, we provide an overview of the cellular and molecular aspects of stress responses in the root stem cell niche.

## Teaser

Environmental stress preferentially induces death of stem cells and their early descendants, which temporally stimulates the division of quiescent center cells but restricts or stops stem cell division.

## Introductionroot stem cell niche structure and dynamics

The root apical meristem (RAM), consisting of proliferating cells at the root tip, maintains root growth and development throughout the plant life cycle (reviewed by Jiang and Feldman, 2005). The RAM harbors a stem cell niche, the specific microenvironment that provides signals that block cell differentiation (reviewed by Laux, 2003; Stahl and Simon, 2005; Aichinger *et al.*, 2012; Perilli *et al.*, 2012). Stem cells (initials) in the root are maintained around a group of mitotically inert quiescent center (QC) cells. The QC of angiosperms varies in size among different plant species, from as few as four cells in Arabidopsis (*Arabidopsis thaliana*) to hundreds of cells in maize (*Zea mays*) (Clowes, 1956, 1958; Dolan *et al.*, 1993).

**Fig. 1.**
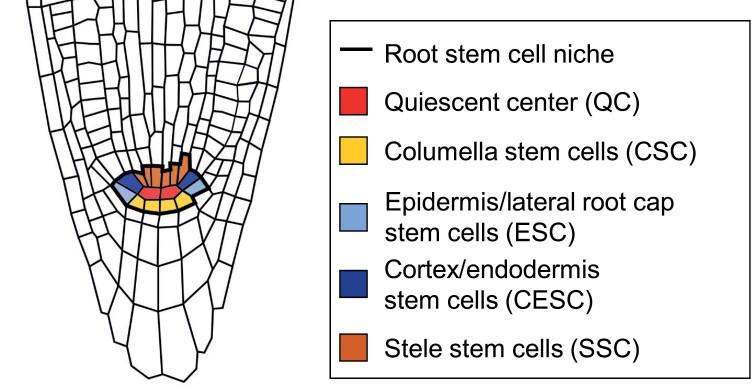
The root stem cell niche in Arabidopsis.

Mitotically active initials, capable of unlimited self-renewal and giving rise to differentiating descendants, circumscribe the QC. Figure 1 shows the classical stem cell niche architecture of *A. thaliana*. Stele stem cells (SSCs) located proximally to the QC generate the stele; stem cells lateral to the QC (CESCs) form the endodermis and cortex, with adjacent stem cells forming the epidermis and lateral root cap (ESCs); and columella stem cells (CSCs) are located below the QC (Dolan *et al.*, 1993). Root stem cells divide asymmetrically (formative cell division), giving rise to a new stem cell plus a daughter cell that differentiates after a limited number of symmetric cell divisions (Stahl and Simon, 2005; De Smet and Beeckman, 2011). CESCs show a division pattern comprising first anticlinal symmetric and second periclinal asymmetric divisions. ESCs undergo two variants of formative cell division: they first divide periclinally, producing a new ESC and a lateral root cell daughter cell; the new ESC then divides anticlinally, producing the epidermis daughter cell (Willemsen *et al.*, 2008; De Smet and Beeckman, 2011).

Experiments with QC laser ablation (Xu *et al.*, 2006) showed that positional information rather than QC cell identity specifies the location and size of the stem cell niche. Thus, the structure of the stem cell niche changes during root development despite stereotypical cell division patterns. In some plants, such as *Sinapis alba*, *Vicia faba*, and *Malva sylvestris*, the QC is absent from the root apex at germination and is organized later during root growth (Clowes, 1958, 1961, 1978). In other plants, including Arabidopsis, the QC is specified during embryogenesis but can be quickly restored upon injury after germination. The duration of the QC mitotic cycle is long but not infinite (Rahni and Birnbaum, 2019). QC daughter cells usually become CSCs (Cruz-Ramirez *et al*., 2013); however, clonal analysis shows that QC cells can potentially replace all stem cells in the meristem (Kidner *et al.*, 2000). Cell divisions in the QC accelerate upon meristem aging, disturbing the stem cell niche structure (Timilsina *et al.*, 2019; Wein *et al.*, 2020). It is unclear whether meristem aging is part of an inherent developmental program or is the consequence of accumulating stress.

## Specific cellular stress responses in the root stem cell niche

**Fig. 2.**
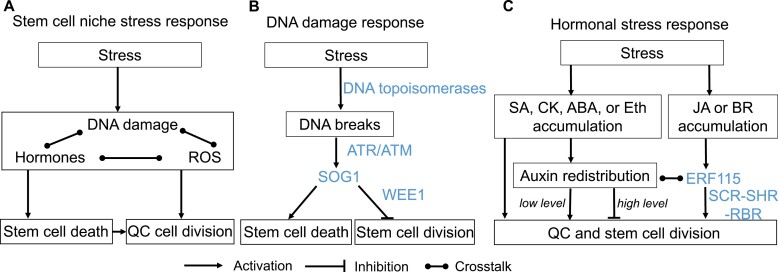
Pathways of the stem cell niche response to stress. The summary (A) and the details of DNA damage (B) and hormonal (C) stress responses. The processes are in boxes; the proteins are colored blue. SA, salicylic acid; CK, cytokinins; JA, jasmonic acid; ABA, abscisic acid; BR, brassinosteroids; Eth, ethylene; ROS, reactive oxygen species.

Upon severe or prolonged stress, the stem cell niche shows two specific cellular responses: activation of QC cell divisions and death of root stem cells (Fig. 2A). Clowes described QC activation upon high dosage irradiation in the middle of the 20th century (Clowes, 1959, 1963). However, the specific vulnerability of root stem cells and their programmed cell death (PCD) as a result of various stresses has been discovered only recently (Fulcher and Sablowski, 2009; Furukawa *et al.*, 2010; Heyman *et al.*, 2013; Hong *et al.*, 2017).

DNA damage-mediated death of root stem cells is triggered by UVB and γ irradiation (Furukawa *et al.*, 2010), X-rays, and radiomimetic drugs such as bleomycin and zeocin (Fulcher and Sablowski, 2009; Heyman *et al.*, 2013). Notably, the PCD response of the root stem cells is cell type specific. SSCs are especially prone to entering the PCD pathway (Fulcher and Sablowski, 2009; Heyman *et al.*, 2013; Cahner *et al*., 2020). In addition to SSCs, high-concentration, long-term (24 h) zeocin treatment kills CSCs and QC cells (Fulcher and Sablowski, 2009); bleomycin also triggers death of CSCs (Cahner *et al*., 2020). Chilling stress induces root stem cell death; however, in most cases, columella stem cell daughters are sacrificed to ensure survival of stem cells (Hong *et al.*, 2017).

In contrast to stem cells, QC cells are highly tolerant of DNA-damaging agents, dying only after exposure to acute stress (Fulcher and Sablowski, 2009; Furukawa *et al.*, 2010). Instead, stress signals activate the cell division machinery in the QC, accelerating the cell cycle (reviewed by Heyman *et al.*, 2014). Thus, the QC serves as a reservoir of cells able to restore root growth in the case of significant damage. Mitotic activation of the QC occurs following root cap cutting (Jiang *et al.*, 2003; Ivanov *et al.*, 2011; Bystrova *et al.*, 2015), chilling stress (Clowes and Stewart, 1967; Barlow and Rathfelder, 1985), flooding-induced hypoxia (Mira *et al.*, 2020), lead-induced toxicity stress (Kozhevnikova *et al.*, 2007), and heat exposure (Clowes and Wadekar, 1989; Kidner *et al.*, 2000; Heyman *et al.*, 2013).

Sacrificing root stem cells undergoing PCD allows the RAM to survive severe stress (Fulcher and Sablowski, 2009). A plausible role for the stem cell death response is maintaining the genetic material of rapidly dividing cells undamaged in order to sustain tissue patterning. It is likely that for symplastically growing plant tissues, either slowly dividing QC cells or dedifferentiated tissues are best able to replenish stem cells with compromised DNA.

Below, we discuss the mechanisms that provoke stress-induced changes in stem cell niche activity and help this region withstand unfavorable conditions (Fig. 2).

## Mechanisms behind root stem cell susceptibility to DNA damage

Accumulating evidence suggests that severe stress leads to DNA fragmentation in root stem cells and their early descendants (Fulcher and Sablowski, 2009; Furukawa *et al.*, 2010; Mironova and Xu, 2019). DNA breaks in stem cells cause DNA replication stress and are particularly disruptive when a cell undergoes mitosis, leading to chromosomal aberrations and mutations. The root stem cell-specific DNA replication stress mechanism is associated with DNA topoisomerases (Zhang *et al.*, 2016) (Fig. 2B). DNA TOPOISOMERASE1 (TOP1) is essential for the survival of SSCs, which appear to be particularly sensitive to torsional stress during DNA replication. DNA topoisomerases relax DNA supercoils by introducing temporary single- or double-strand breaks (Champoux, 2001).

Two cell cycle checkpoint kinases, ATAXIA TELANGIECTASIA MUTATED (ATM) and ATM AND RAD3-RELATED (ATR), transmit DNA damage signals in plant cells (Abraham, 2001; Garcia *et al.*, 2003; Culligan *et al.*, 2004). ATM responds to double-strand DNA breaks (Bensimon *et al.*, 2011), while ATR transmits signals about single-strand DNA breaks (Flynn and Zou, 2011). The NAC family transcription factor SUPPRESSOR OF GAMMA RESPONSE1 (SOG1) is phosphoactivated by ATM to trigger the DNA damage response (Yoshiyama *et al.*, 2013). In plant stem cells, SOG1 governs the key cell death pathway induced by high-intensity UVB radiation, X-rays, and radiomimetic drugs (Fulcher and Sablowski, 2009; Furukawa *et al.*, 2010). The SOG1 downstream cascade that triggers cell death remains largely unknown; however, many direct targets of SOG1 have been discovered recently (Ogita *et al.*, 2018; Ryu *et al.*, 2019).

Several mechanisms that restrict uncontrolled stem cell death exist. The MEDIATOR (MED) complex subunit (MED18) protects root meristem cells from DNA damage-mediated cell death; *med18* mutants show spontaneous death of vascular initials and their daughters (Raya-González *et al.*, 2018). Besides cell death, the SOG1-dependent pathway also mediates stem cell survival following stress-induced DNA damage by inhibiting cell cycle progression at the G_2_/M checkpoint (Furukawa *et al.*, 2010; Yoshiyama *et al.*, 2013). Temporal cell cycle arrest prevents the mitotic catastrophe that might happen if DNA is left unrepaired. ATR regulates cell cycle arrest at the G_2_/M checkpoint in response to irradiation (Culligan *et al.*, 2004) and aluminum (Rounds and Larsen, 2008; Sjogren *et al.*, 2015). Temporal inhibition of CSC division occurs upon chilling stress; when it finally divides, the CSC daughter tends to undergo PCD (Hong *et al.*, 2017).

SOG1 targets numerous genes responsible for cell cycle regulation at the G_2_/M transition, including cyclin-dependent kinase (CDK) inhibitor genes *KIP-RELATED PROTEIN 6* (*KRP6*), *SIAMESE-RELATED* (*SMR*) *SMR4*, *5*, *7*, and *WEE1* (Ogita *et al.*, 2018). KRP and SMR bind to CDK–cyclin complexes and inhibit their kinase activity (Van Leene *et al.*, 2010; Yi *et al.*, 2014). WEE1 kinase phosphorylates and inactivates the CDKs that mediate the G_2_/M phase cell cycle transition (De Schutter *et al.*, 2007). Direct targets of SOG1, homologous transcription factor-coding genes *ANAC044* and *ANAC085*, play a crucial role in G_2_/M cell cycle arrest upon DNA damage response (Takahashi *et al.*, 2019). Similar to *sog1* mutants, the stem cell niche of *anac044* and *anac085* mutant plants is tolerant to DNA-damaging agents. However, the increased tolerance of *anac044/085* mutants is associated with G_2_/M checkpoint control, rather than through increased DNA repair. ANAC044/ANAC085 are essential in regulating protein accumulation of the R1R2R3-type Myb transcription factors (MYB3R), which mediate G_2_/M-specific genes expression both positively and negatively (Okumura *et al.*, 2021). Notably, *myb3r3* and *myb3r5* mutants, which cannot induce G_2_/M arrest upon DNA damage response, also show less zeocin-induced stem cell death (Chen *et al.*, 2017). Interestingly, *ANAC044*/*ANAC085* have been implicated in the regulation of a stress-specific function, mediating heat stress, but not osmotic stress-induced G_2_/M arrest (Takahashi *et al.*, 2019).

## DNA damage-activated backup plan: QC cell divisions

DNA damage-induced cell death activates regeneration programs in the stem cell niche, consequently inducing divisions in the QC (Heyman *et al.*, 2013, 2014). The transcription factor ETHYLENE RESPONSE FACTOR 115 (ERF115) is the master regulator of damage-induced regenerative processes. Under non-stress conditions, ERF115 is only expressed in dividing QC cells, serving as a rate-limiting regulator of divisions in the QC (Heyman *et al.*, 2013). Under stress, ERF115 is activated in the QC, around dead cells, and in the endodermis. This activation induces restorative cell divisions (Heyman *et al.*, 2016; Zhou *et al.*, 2019; Canher *et al.*, 2020). ERF115-dependent activation of QC cell division is detected in response to heat stress, wounding, and nematode infection (Heyman *et al.*, 2013; Zhou *et al.*, 2019).

*ERF115* is not a direct target of SOG1; rather, it is induced in the stem cell niche as a result of SOG1-dependent PCD (Johnson *et al.*, 2018). ERF109, a close homolog of ERF115, is rapidly induced after cell ablation and triggers ectopic ERF115 expression around the dead cells, activating regeneration processes in the meristem (Heyman *et al.*, 2016; Zhou *et al.*, 2019). ERF115 recruits the regulatory circuit SCARECROW (SCR)–SHORT ROOT (SHR)–RETINOBLASTOMA-RELATED (RBR), which guides asymmetric cell division of root stem cells (Paquette and Benfey, 2005; Cruz-Ramírez *et al.*, 2012, 2013). ERF115 binds to and inhibits RBR activity (Zhou *et al.*, 2019). This blocks RBR–SCR interaction and allows QC cell division. ERF115 also forms a heterodimer with PHYTOCHROME A SIGNAL TRANSDUCTION1 (PAT1) to mediate restorative cell divisions, for example stem cell niche recovery upon root tip excision (Heyman *et al.*, 2016). One putative PAT1–ERF115 target is WOUND INDUCED DEDIFFERENTIATION1 (WIND1), a key factor promoting plant cell dedifferentiation (Iwase *et al.*, 2011; Heyman *et al.*, 2016). Co-expression of PAT1 with ERF115 hyperinduces WIND1. Another potential target of ERF115 is the AUXIN RESPONSE FACTOR 5 (ARF5), a major regulator of auxin signaling in root development (Canher *et al.*, 2020). The *ARF5* upstream region possesses ERF115-binding sites, and its expression corresponds with the level of ERF115.

## Not merely a by-product: the crucial role of ROS in the stem cell niche

Stress-induced changes in stem cell niche activity also rely on changes in the distribution of reactive oxygen species (ROS). A by-product of aerobic metabolism, ROS are highly reactive molecules that can induce DNA damage, protein oxidation, and lipid peroxidation (reviewed by Gill and Tuteja, 2010; Huang *et al.*, 2019). ROS exist in ionic and molecular states: ionic forms include hydroxyl radicals (OH·) and superoxide anions (O_2_· ^–^); molecular forms include hydrogen peroxide (H_2_O_2_) and singlet oxygen (^1^O_2_). An antioxidant system consists of ROS scavenger enzymes and non-enzymatic low molecular weight metabolites [ascorbic acid (ASC), reduced glutathione (GSH), carotenoids, flavonoids, and proline] that counteract uncontrolled oxidation (Schafer and Buettner, 2001; Conklin and Barth, 2004; reviewed in Jiang and Feldman, 2005). Cellular redox potential is determined by the contribution of different redox couples and ROS, and is controlled by a delicate balance between ROS production and scavenging (reviewed by Lee *et al.*, 2019).

Cellular redox potential plays a critical role in regulating cell proliferation. In the stem cell niche, QC cells have a more highly oxidized status than surrounding stem cells (Jiang *et al.*, 2003; Jiang and Feldman, 2005), which is essential for maintenance of QC dormancy (reviewed by Huang *et al.*, 2019; Eljebbawi *et al.*, 2020). Indeed, *miao* mutants deficient in the plastid-localized GR2 enzyme, which is part of the plant antioxidant system, exhibit a partial loss of QC identity mediated by a perturbed auxin maximum (Yu *et al.*, 2013). Knockout of *VITAMIN C DEFECTIVE 1* (*VTC1*), a rate-limiting gene affecting the quantity of ascorbic acid, results in elevated H_2_O_2_ levels that increase the number of QC cells and periclinal divisions in the root meristem (Kka *et al.*, 2018). It is noteworthy that both reducing the oxidative status of the QC and treating roots with exogenous H_2_O_2_ lead to QC activation (Jiang *et al.*, 2003; Kong *et al.*, 2018).

Stress conditions such as heat, cold, drought, heavy metals, and pathogens rapidly disturb the redox balance by inducing ROS accumulation in plant tissues (Lee *et al.*, 2012; Kawarazaki *et al.*, 2013; Kim and Hwang, 2014; Zhao *et al.*, 2018). While ROS bursts under severe stress conditions can cause intense oxidative stress, sometimes leading to whole-organ death, under moderate stress conditions ROS activate signaling pathways that trigger adaptive stress response programs. A well-described example of ROS-mediated damage and adaptation in the stem cell niche is flooding-induced hypoxia. Maintaining well-balanced, low levels of ROS is crucial for root meristem survival under hypoxic conditions (Sasidharan *et al.*, 2018). Hypoxia-induced accumulation of ROS and nitric oxide (NO) causes QC cell division and death of meristematic root cells (Mira *et al.*, 2016). A decline in either O_2_ or NO leads to expression of core hypoxia genes and hypoxia acclimation (Gibbs *et al.*, 2011, 2018).

## Pivotal role of auxin in root stem cell niche maintenance

The auxin concentration maximum defines QC identity and maintains stem cell niche integrity (Jiang and Feldman, 2005). Auxin biosynthesis, conjugation, oxidation, and, most critically, transportation networks work together to generate and support the auxin maximum in the stem cell niche. Maintaining a dynamic balance in auxin patterning helps plants withstand the rigors of environmental stress.

Environmental cues commonly affect root growth plasticity by influencing auxin biosynthesis, transport, and signaling (Pierik and Testerik, 2014; Korver *et al.*, 2018). Despite different stresses having specific targets in these auxin pathways, sometimes outside the meristem, all of them potentially influence stem cell niche activity to some extent. Stress-induced messages that affect the shoots are delivered to the root meristem by long-distance auxin transport; short-distance auxin transport consequently alters auxin levels in the root stem cell niche. For example, iron deficiency decreases auxin transport from shoots to roots in rice (Sun *et al.*, 2017). Mathematical modeling suggests that the rate of auxin inflow into the root meristem is a critical parameter affecting the maintenance of the auxin maximum (Mironova *et al.*, 2010).

**Fig. 3.**
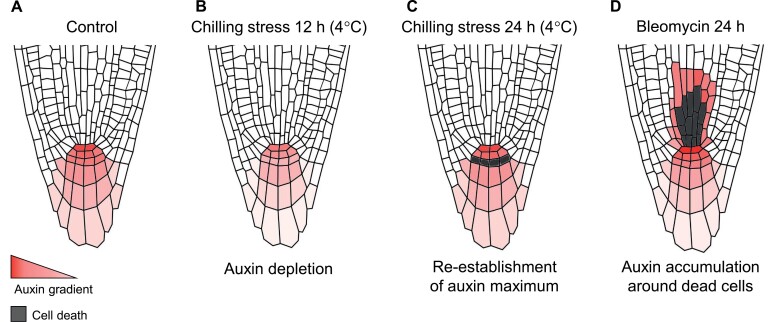
Auxin dynamics in the stem cell niche in response to stress. (A) Auxin maximum in the QC maintains stem cell niche integrity. (B) Auxin levels in the meristem are depleted in response to different stresses (e.g. chilling stress at 4 °C; Hong *et al.*, 2017), resulting in loss of QC identity and precocious divisions in the stem cell niche. (C) Re-establishment of the auxin maximum in the QC occurs after chilling stress-specific CSC daughter death (Hong *et al.*, 2017). (D) Bleomycin-induced cell death of SSCs and their daughters causes auxin accumulation around the wound, activating restorative cell divisions (Canher *et al.*, 2020).

QC activation and root meristem exhaustion upon severe stress often correspond to depletion of auxin in the stem cell niche (Fig. 3). A decrease in activity of the auxin response marker DR5 might also indicate the maladaptive status and vulnerability of the stem cell niche to stress. Low-potassium (K^+^) conditions slightly decrease DR5 signal in the QC, corresponding to an acceleration of QC cell division compared to control conditions; this phenotype is greatly enhanced in the *kup9* mutant defective in K^+^ and auxin efflux from the endoplasmic reticulum (Zhang *et al.*, 2020). Chilling stress causes a decrease in DR5 activity in the QC, contributing to induction of CSC division and CSC daughter death (Hong *et al.*, 2017) (Fig. 3A, B). Generally, a decrease in QC-localized DR5 signal corresponds strongly to misexpression of auxin transporters from PIN-FORMED or AUX/LAX families.

Local auxin biosynthesis in the stem cell niche has less influence on QC maintenance than PIN-mediated transport, but helps the plant to rapidly enhance auxin levels in the root tip upon stress. Expression of the *TRYPTOPHAN AMINOTRANSFERASE OF ARABIDOPSIS 1* (*TAA1*) gene encoding an auxin biosynthesis enzyme is enhanced upon Al exposure (L. Yang *et al.*, 2014), leading to auxin accumulation in the root tip. Auxin biosynthesis via TAA/TAR enzymes is essential for the root meristem response to the stress hormone ethylene (Brumos *et al.*, 2018). Enhanced auxin biosynthesis rates are also observed at the site of root tip injury (Matosevich *et al.*, 2020).

Dead cells affect auxin patterning via disruption of PIN-mediated auxin transport routes in the meristem (Canher *et al.*, 2020). Bleomycin-mediated death of SSCs leads to rapid accumulation of auxin around the dead cells without activating auxin biosynthesis (Fig. 3D). Auxin accumulation in the endodermis promotes replenishment of SSCs via an ERF115-dependent pathway. As another example, DNA damage-induced CSC daughter death partially blocks lateral auxin redistribution in the columella, leading to auxin accumulation in the QC upon chilling stress (Hong *et al.*, 2017). Intriguingly, plants with sacrificed CSC daughters and boosted auxin levels in the QC not only recover faster from chilling stress but also withstand accompanying freezing, drought, and even genotoxic zeocin treatments better than those plants not sacrificing these cells. Moreover, auxin protects stem cells against zeocin-induced cell death (Hong *et al.*, 2017).

## The stem cell niche does not live by auxin alone: other plant hormones

Although a change in auxin patterning precedes division of the QC cells (Jiang *et al.*, 2003), the QC is activated by exposure to ethylene (Ortega-Martinez *et al*., 2007), jasmonic acid (JA) (Zhou *et al.*, 2019), salicylic acid (SA) (Pasternak *et al.*, 2019), cytokinin (Zhang *et al.*, 2013), and brassinosteroids (BRs) (Lozano-Elena *et al.*, 2018) (Fig. 2C). Cytokinin negatively regulates the auxin influx carrier LAX2 in the meristem, with the *lax2* mutant showing reduced auxin levels in the stem cell niche and ectopic divisions in the QC (Zhang *et al.*, 2013). The morphogenetic role of low-level exogenous SA is determined by its dose-dependent control of auxin transport and biosynthesis (Pasternak *et al.*, 2019). Exposure to low-level SA leads to stem cell niche enlargement via activation of PIN1 and TAA1 and inhibition of PIN2 and PIN7. Another explanation for hormone-induced division of QC cells is precocious RAM aging. Prolonged treatments with relatively high concentrations of exogenous hormones are typically used to induce division of QC cells, which might be stressful for the stem cell niche. QC cell divisions occur more frequently in aging plants than in younger plants (Timilsina *et al.*, 2019).

The response and acclimation of the root stem cell niche to stress also rely on hormone-specific effects that are independent of auxin. Restricted ethylene diffusion in compacted soil or upon flooding leads to ethylene accumulation in the root tip, which helps the meristem to adapt to the stress (Hartman *et al.*, 2019; Pandey *et al.*, 2021). Ethylene signaling activates NO scavenging by PHYTOGLOBIN1 (PGB1), which is essential for acclimating the meristem to flooding-induced hypoxia (Hartman *et al.*, 2019). PGB1 reduces NO levels and stabilizes ERFVII transcription factors, which help the stem cell niche to withstand hypoxia. PGB1 is also essential for adaptation to water deficit (Mira *et al.*, 2017).

Abscisic acid (ABA) has a specific role in the stem cell niche, namely maintaining the stem cell niche in a juvenile state and ensuring QC dormancy (Zhang *et al.*, 2010). ABA treatment suppresses cell division in the meristem for long periods without loss of meristem function. At least partially, ABA exerts its role on meristematic activity by modulating auxin transportation and signaling (Zhang *et al.*, 2010; Rowe *et al.*, 2016; Promchuea *et al.*, 2017). However, ABA-mediated production of ROS in mitochondria is also crucial for maintaining stem cell niche activity and the auxin response maximum (Z.B. Yang *et al.*, 2014). Furthermore, SA promotes ROS accumulation in the stem cell niche (Wang *et al.*, 2021).

It is noteworthy that salt stress initiates an increase in ABA and a decrease in BR signaling in the inner tissues; these events are followed by activation of JA and derepression of BR pathways (Geng *et al.*, 2013). These observations indirectly support the idea that the QC is temporally protected under early stress response but later on its cells divide to replenish the damaged cells. BRs recruit the BRI1-EMS-SUPPRESSOR 1 (BES1)–BRASSINOSTEROIDS AT VASCULAR AND ORGANIZING CENTER (BRAVO)–ERF115 signaling module to control QC cell divisions (Vilarrasa-Blasi *et al.*, 2014).

Recent studies demonstrated that JA plays a pivotal role in stem cell niche regeneration (Zhou *et al.*, 2019). Wounding leads to JA accumulation that rapidly induces transcription of *ERF109*. ERF109 stimulates CYCD6;1 expression in the endodermis and QC, and triggers *ERF115* expression in the stele. Methyl jasmonate pre-treatment to induce *ERF115* expression before cell ablation promotes faster replenishment of dead cells. JA and auxin synergistically activate the SCR–SHR–RBR pathway to guide restorative cell divisions when roots are cut, penetrate the soil, or are infected with nematodes.

## Conclusion

Figure 2 summarizes major pathways of stress-induced responses in the root stem cell niche. This roadmap is certainly incomplete, missing multiple condition-specific crosstalk and feedback routes between the major pathways. For example, H_2_O_2_ treatment activates ERF115-mediated QC cell division independently of cell death signaling (Kong *et al.*, 2018); ERF115 enhances auxin signaling via the ARF5/MP transcription factor, and ARF5/MP, in turn, promotes the ERF115 pathway (Canher *et al.*, 2020); and reduction of the QC oxidation status corresponds to auxin depletion (Jiang *et al.*, 2003). DNA damage response, ROS, auxin distribution, the ERF115-mediated cascade, and hormonal signaling are all interconnected, facilitating plant adaptation to numerous adverse conditions. Identifying key components of the root stem cell niche response to stress will help scientists to sustain, select, or bioengineer plants that effectively tolerate particular stresses, thus widening the scope of sustainable agriculture.

## References

[CIT0001] Abraham RT . 2001. Cell cycle checkpoint signaling through the ATM and ATR kinases. Genes & Development 15, 2177–2196. 11544175 10.1101/gad.914401

[CIT0002] Aichinger E , Kornet N, Friedrich T, Laux T. 2012. Plant stem cell niches. Annual Review of Plant Biology 63, 615–636. 10.1146/annurev-arplant-042811-105555 22404469

[CIT0003] Barlow PW , Rathfelder EL. 1985. Cell division and regeneration in primary root meristems of *Zea mays* recovering from cold treatment. Environmental and Experimental Botany 25, 303–314.

[CIT0004] Bensimon A , Aebersold R, Shiloh Y. 2011. Beyond ATM: the protein kinase landscape of the DNA damage response. FEBS Letters 585, 1625–1639. 21570395 10.1016/j.febslet.2011.05.013

[CIT0005] Brumos J , Robles LM, Yun J, Vu TC, Jackson S, Alonso JM, Stepanova AN. 2018. Local auxin biosynthesis is a key regulator of plant development. Developmental Cell 47, 306–318.e5. 30415657 10.1016/j.devcel.2018.09.022

[CIT0006] Bystrova EI , Zhukovskaya NV, Rakitin VJ, Ivanov VB. 2015. Role of ethylene in activation of cell division in quiescent center of excised maize roots. Russian Journal of Developmental Biology 46, 60–64. 26021120

[CIT0007] Canher B , Heyman J, Savina M, et al. 2020. Rocks in the auxin stream: wound-induced auxin accumulation and ERF115 expression synergistically drive stem cell regeneration. Proceedings of the National Academy of Sciences, USA 117, 16667–16677. 10.1073/pnas.2006620117 PMC7368246 32601177

[CIT0008] Champoux JJ . 2001. DNA topoisomerases: structure, function, and mechanism. Annual Review of Biochemistry 70, 369–413. 10.1146/annurev.biochem.70.1.369 11395412

[CIT0009] Chen P , Takatsuka H, Takahashi N, Kurata R, Fukao Y, Kobayashi K, Ito M, Umeda M. 2017. Arabidopsis R1R2R3-Myb proteins are essential for inhibiting cell division in response to DNA damage. Nature Communications 8, 635. 10.1038/s41467-017-00676-4 PMC5608833 28935922

[CIT0010] Clowes FAL . 1956. Localization of nucleic acid synthesis in root meristems. Journal of Experimental Botany 7, 307–312.

[CIT0011] Clowes FAL . 1958. Development of quiescent centres in root meristems. New Phytologist 57, 85–88.

[CIT0012] Clowes FAL . 1959. Reorganization of root apices after irradiation. Annals of Botany 23, 205–210.

[CIT0013] Clowes FAL . 1961. Apical meristems. Oxford: Blackwell Scientific Publications.

[CIT0014] Clowes FAL . 1963. X-irradiation of root meristems. Annals of Botany 27, 344–352.

[CIT0015] Clowes FAL . 1978. Origin of quiescence at the root pole of pea embryos. Annals of Botany 42, 1237–1239.

[CIT0016] Clowes FAL , Stewart HE. 1967. Recovery from dormancy in roots. New Phytologist 66, 115–125.

[CIT0017] Clowes FAL , Wadekar R. 1989. Instability in the root meristem of *Zea mays* during growth. New Phytologist 111, 19–24.

[CIT0018] Conklin PL , Barth C. 2004. Ascorbic acid, a familiar small molecule intertwined in the response of plants to ozone, pathogens, and the onset of senescence. Plant, Cell & Environment 27, 959–970.

[CIT0019] Cruz-Ramírez A , Díaz-Triviño S, Blilou I, et al. 2012. A bistable circuit involving SCARECROW–RETINOBLASTOMA integrates cues to inform asymmetric stem cell division. Cell 150, 1002–1015. 22921914 10.1016/j.cell.2012.07.017 PMC3500399

[CIT0020] Cruz-Ramírez A , Díaz-Triviño S, Wachsman G, et al. 2013. A SCARECROW–RETINOBLASTOMA protein network controls protective quiescence in the Arabidopsis root stem cell organizer. PLoS Biology 11, e1001724. 24302889 10.1371/journal.pbio.1001724 PMC3841101

[CIT0021] Culligan K , Tissier A, Britt A. 2004. ATR regulates a G_2_-phase cell-cycle checkpoint in *Arabidopsis thaliana*. The Plant Cell 16, 1091–1104. 15075397 10.1105/tpc.018903 PMC423202

[CIT0022] De Schutter K , Joubès J, Cools T, et al. 2007. Arabidopsis WEE1 kinase controls cell cycle arrest in response to activation of the DNA integrity checkpoint. The Plant Cell 19, 211–225. 17209125 10.1105/tpc.106.045047 PMC1820959

[CIT0023] De Smet I , Beeckman T. 2011. Asymmetric cell division in land plants and algae: the driving force for differentiation. Nature Reviews. Molecular Cell Biology 12, 177–188. 21346731 10.1038/nrm3064

[CIT0024] Dolan L , Janmaat K, Willemsen V, Linstead P, Poethig S, Roberts K, Scheres B. 1993. Cellular organisation of the *Arabidopsis thaliana* root. Development 119, 71–84. 8275865 10.1242/dev.119.1.71

[CIT0025] Eljebbawi A , Guerrero YDCR, Dunand C, Estevez JM. 2020. Highlighting reactive oxygen species as multitaskers in root development. iScience 24, 101978. 33490891 10.1016/j.isci.2020.101978 PMC7808913

[CIT0026] Flynn RL , Zou L. 2011. ATR: a master conductor of cellular responses to DNA replication stress. Trends in Biochemical Sciences 36, 133–140. 20947357 10.1016/j.tibs.2010.09.005 PMC3024454

[CIT0027] Fulcher N , Sablowski R. 2009. Hypersensitivity to DNA damage in plant stem cell niches. Proceedings of the National Academy of Sciences, USA 106, 20984–20988. 10.1073/pnas.0909218106 PMC2791609 19933334

[CIT0028] Furukawa T , Curtis MJ, Tominey CM, Duong YH, Wilcox BW, Aggoune D, Hays JB, Britt AB. 2010. A shared DNA-damage–response pathway for induction of stem-cell death by UVB and by gamma irradiation. DNA Repair 9, 940–948. 20634150 10.1016/j.dnarep.2010.06.006

[CIT0029] Garcia V , Bruchet H, Camescasse D, Granier F, Bouchez D, Tissier A. 2003. AtATM is essential for meiosis and the somatic response to DNA damage in plants. The Plant Cell 15, 119–132. 12509526 10.1105/tpc.006577 PMC143473

[CIT0030] Geng Y , Wu R, Wee CW, Xie F, Wei X, Chan PM, Tham C, Duan L, Dinneny JR. 2013. A spatio-temporal understanding of growth regulation during the salt stress response in Arabidopsis. The Plant cell 25, 2132–2154. 23898029 10.1105/tpc.113.112896 PMC3723617

[CIT0031] Gibbs DJ , Lee SC, Isa NM, et al. 2011. Homeostatic response to hypoxia is regulated by the N-end rule pathway in plants. Nature 479, 415–418. 22020279 10.1038/nature10534 PMC3223408

[CIT0032] Gibbs DJ , Tedds HM, Labandera AM, et al. 2018. Oxygen-dependent proteolysis regulates the stability of angiosperm polycomb repressive complex 2 subunit VERNALIZATION 2. Nature Communications 9, 5438. 10.1038/s41467-018-07875-7 PMC6303374 30575749

[CIT0033] Gill SS , Tuteja N. 2010. Reactive oxygen species and antioxidant machinery in abiotic stress tolerance in crop plants. Plant Physiology and Biochemistry 48, 909–930. 20870416 10.1016/j.plaphy.2010.08.016

[CIT0034] Hartman S , Liu Z, van Veen H, et al. 2019. Ethylene-mediated nitric oxide depletion pre-adapts plants to hypoxia stress. Nature Communications 10, 4020. 10.1038/s41467-019-12045-4 PMC6728379 31488841

[CIT0035] Heyman J , Cools T, Canher B, et al. 2016. The heterodimeric transcription factor complex ERF115–PAT1 grants regeneration competence. Nature Plants 2, 16165. 27797356 10.1038/nplants.2016.165

[CIT0036] Heyman J , Cools T, Vandenbussche F, et al. 2013. ERF115 controls root quiescent center cell division and stem cell replenishment. Science 342, 860–863. 24158907 10.1126/science.1240667

[CIT0037] Heyman J , Kumpf RP, De Veylder L. 2014. A quiescent path to plant longevity. Trends in Cell Biology 24, 443–448. 24704103 10.1016/j.tcb.2014.03.004

[CIT0038] Hong JH , Savina M, Du J, Devendran A, Kannivadi Ramakanth K, Tian X, Sim WS, Mironova VV, Xu J. 2017. A sacrifice-for-survival mechanism protects root stem cell niche from chilling stress. Cell 170, 102–113.e14. 28648662 10.1016/j.cell.2017.06.002

[CIT0039] Huang H , Ullah F, Zhou DX, Yi M, Zhao Y. 2019. Mechanisms of ROS regulation of plant development and stress responses. Frontiers in Plant Science 10, 800. 31293607 10.3389/fpls.2019.00800 PMC6603150

[CIT0040] Ivanov VB , Bystrova EI, Mesenko MM, Kotov AA. 2011. Cell division activation in the quiescent center of excised maize root tip. Russian Journal of Developmental Biology 42, 311–316. 22145304

[CIT0041] Iwase A , Ohme-Takagi M, Sugimoto K. 2011. WIND1: a key molecular switch for plant cell dedifferentiation. Plant Signaling & Behavior 6, 1943–1945. 22112447 10.4161/psb.6.12.18266 PMC3337183

[CIT0042] Jiang K , Feldman LJ. 2005. Regulation of root apical meristem development. Annual Review of Cell and Developmental Biology 21, 485–509. 10.1146/annurev.cellbio.21.122303.114753 16212504

[CIT0043] Jiang K , Meng YL, Feldman LJ. 2003. Quiescent center formation in maize roots is associated with an auxin-regulated oxidizing environment. Development 130, 1429–1438. 12588857 10.1242/dev.00359

[CIT0044] Johnson RA , Conklin PA, Tjahjadi M, Missirian V, Toal T, Brady SM, Britt AB. 2018. SUPPRESSOR OF GAMMA RESPONSE1 links DNA damage response to organ regeneration. Plant Physiology 176, 1665–1675. 29222192 10.1104/pp.17.01274 PMC5813563

[CIT0045] Kawarazaki T , Kimura S, Iizuka A, Hanamata S, Nibori H, Michikawa M, Imai A, Abe M, Kaya H, Kuchitsu K. 2013. A low temperature-inducible protein AtSRC2 enhances the ROS-producing activity of NADPH oxidase AtRbohF. Biochimica et Biophysica Acta 1833, 2775–2780. 23872431 10.1016/j.bbamcr.2013.06.024

[CIT0046] Kidner C , Sundaresan V, Roberts K, Dolan L. 2000. Clonal analysis of the Arabidopsis root confirms that position, not lineage, determines cell fate. Planta 211, 191–199. 10945213 10.1007/s004250000284

[CIT0047] Kim DS , Hwang BK. 2014. An important role of the pepper phenylalanine ammonia-lyase gene (PAL1) in salicylic acid-dependent signalling of the defence response to microbial pathogens. Journal of Experimental Botany 65, 2295–2306. 24642849 10.1093/jxb/eru109 PMC4036500

[CIT0048] Kka N , Rookes J, Cahill D. 2018. The influence of ascorbic acid on root growth and the root apical meristem in *Arabidopsis thaliana*. Plant Physiology and Biochemistry 129, 323–330. 29929127 10.1016/j.plaphy.2018.05.031

[CIT0049] Kong X , Tian H, Yu Q, et al. 2018. PHB3 maintains root stem cell niche identity through ROS-responsive AP2/ERF transcription factors in Arabidopsis. Cell Reports 22, 1350–1363. 29386120 10.1016/j.celrep.2017.12.105

[CIT0050] Korver RA , Koevoets IT, Testerink C. 2018. Out of shape during stress: a key role for auxin. Trends in Plant Science 23, 783–793. 29914722 10.1016/j.tplants.2018.05.011 PMC6121082

[CIT0051] Kozhevnikova AD , Seregin IV, Bystrova EI. et al. 2007. Effects of heavy metals and strontium on division of root cap cells and meristem structural organization. Russian Journal of Plant Physiology 54, 257–266.

[CIT0052] Laux T . 2003. The stem cell concept in plants: a matter of debate. Cell 113, 281–283. 12732137 10.1016/s0092-8674(03)00312-x

[CIT0053] Lee S , Seo PJ, Lee HJ, Park CM. 2012. A NAC transcription factor NTL4 promotes reactive oxygen species production during drought-induced leaf senescence in Arabidopsis. The Plant Journal 70, 831–844. 22313226 10.1111/j.1365-313X.2012.04932.x

[CIT0054] Lee Y . 2019. Redox control on stem cell fate and maintenance in the root. Journal of Plant Biology 62, 320–328.

[CIT0055] Lozano-Elena F , Planas-Riverola A, Vilarrasa-Blasi J, Schwab R, Caño-Delgado AI. 2018. Paracrine brassinosteroid signaling at the stem cell niche controls cellular regeneration. Journal of Cell Science 131, jcs204065. 29242230 10.1242/jcs.204065 PMC5818034

[CIT0056] Matosevich R , Cohen I, Gil-Yarom N, Modrego A, Friedlander-Shani L, Verna C, Scarpella E, Efroni I. 2020. Local auxin biosynthesis is required for root regeneration after wounding. Nature Plants 6, 1020–1030. 32747761 10.1038/s41477-020-0737-9

[CIT0057] Mira MM , El-Khateeb EA, Gaafar RM, Igamberdiev AU, Hill RD, Stasolla C. 2020. Stem cell fate in hypoxic root apical meristems is influenced by phytoglobin expression. Journal of Experimental Botany 71, 1350–1362. 31541257 10.1093/jxb/erz410

[CIT0058] Mira MM , Hill RD, Stasolla C. 2016. Phytoglobins improve hypoxic root growth by alleviating apical meristem cell death. Plant Physiology 172, 2044–2056. 27702845 10.1104/pp.16.01150 PMC5100795

[CIT0059] Mira MM , Huang S, Kapoor K, Hammond C, Hill RD, Stasolla C. 2017. Expression of Arabidopsis class 1 phytoglobin (AtPgb1) delays death and degradation of the root apical meristem during severe PEG-induced water deficit. Journal of Experimental Botany 68, 5653–5668. 29059380 10.1093/jxb/erx371 PMC5853930

[CIT0060] Mironova V , Xu J. 2019. A single-cell view of tissue regeneration in plants. Current Opinion in Plant Biology 52, 149–154. 31655397 10.1016/j.pbi.2019.09.003

[CIT0061] Mironova VV , Omelyanchuk NA, Yosiphon G, Fadeev SI, Kolchanov NA, Mjolsness E, Likhoshvai VA. 2010. A plausible mechanism for auxin patterning along the developing root. BMC Systems Biology 4, 98. 20663170 10.1186/1752-0509-4-98 PMC2921385

[CIT0062] Ogita N , Okushima Y, Tokizawa M, et al. 2018. Identifying the target genes of SUPPRESSOR OF GAMMA RESPONSE 1, a master transcription factor controlling DNA damage response in Arabidopsis. The Plant Journal 94, 439–453. 29430765 10.1111/tpj.13866

[CIT0063] Okumura T , Nomoto Y, Kobayashi K, Suzuki T, Takatsuka H, Ito M. 2021. MYB3R-mediated active repression of cell cycle and growth under salt stress in *Arabidopsis thaliana*. Journal of Plant Research 134, 261–277. 33580347 10.1007/s10265-020-01250-8

[CIT0064] Ortega-Martínez O , Pernas M, Carol RJ, Dolan L. 2007. Ethylene modulates stem cell division in the *Arabidopsis thaliana* root. Science 317, 507–510. 17656722 10.1126/science.1143409

[CIT0065] Pandey BK , Huang G, Bhosale R, et al. 2021. Plant roots sense soil compaction through restricted ethylene diffusion. Science 371, 276–280. 33446554 10.1126/science.abf3013

[CIT0066] Paquette AJ , Benfey PN. 2005. Maturation of the ground tissue of the root is regulated by gibberellin and SCARECROW and requires SHORT-ROOT. Plant Physiology 138, 636–640. 15955927 10.1104/pp.104.058362 PMC1150384

[CIT0067] Pasternak T , Groot EP, Kazantsev FV, Teale W, Omelyanchuk N, Kovrizhnykh V, Palme K, Mironova VV. 2019. Salicylic acid affects root meristem patterning via auxin distribution in a concentration-dependent manner. Plant Physiology 180, 1725–1739. 31036755 10.1104/pp.19.00130 PMC6752920

[CIT0068] Perilli S , Di Mambro R, Sabatini S. 2012. Growth and development of the root apical meristem. Current Opinion in Plant Biology 15, 17–23. 22079783 10.1016/j.pbi.2011.10.006

[CIT0069] Pierik R , Testerink C. 2014. The art of being flexible: how to escape from shade, salt, and drought. Plant Physiology 166, 5–22. 24972713 10.1104/pp.114.239160 PMC4149730

[CIT0070] Promchuea S , Zhu Y, Chen Z, Zhang J, Gong Z. 2017. ARF2 coordinates with PLETHORAs and PINs to orchestrate ABA-mediated root meristem activity in Arabidopsis . Journal of Integrative Plant Biology 59, 30–43. 28074634 10.1111/jipb.12506

[CIT0071] Rahni R , Birnbaum KD. 2019. Week-long imaging of cell divisions in the Arabidopsis root meristem. Plant Methods 15, 30. 30988691 10.1186/s13007-019-0417-9 PMC6446972

[CIT0072] Raya-González J , Oropeza-Aburto A, López-Bucio JS, Guevara-García ÁA, de Veylder L, López-Bucio J, Herrera-Estrella L. 2018. MEDIATOR18 influences Arabidopsis root architecture, represses auxin signaling and is a critical factor for cell viability in root meristems. The Plant Journal 96, 895–909. 30270572 10.1111/tpj.14114

[CIT0073] Rounds MA , Larsen PB. 2008. Aluminum-dependent root-growth inhibition in Arabidopsis results from AtATR-regulated cell-cycle arrest. Current Biology 18, 1495–1500. 18835170 10.1016/j.cub.2008.08.050

[CIT0074] Rowe JH , Topping JF, Liu J, Lindsey K. 2016. Abscisic acid regulates root growth under osmotic stress conditions via an interacting hormonal network with cytokinin, ethylene and auxin. New Phytologist 211, 225–239. 10.1111/nph.13882 PMC4982081 26889752

[CIT0075] Ryu TH , Go YS, Choi SH, Kim JI, Chung BY, Kim JH. 2019. SOG1-dependent NAC103 modulates the DNA damage response as a transcriptional regulator in Arabidopsis. The Plant Journal 98, 83–96. 30554433 10.1111/tpj.14201

[CIT0076] Sasidharan R , Hartman S, Liu Z, Martopawiro S, Sajeev N, van Veen H, Yeung E, Voesenek LACJ. 2018. Signal dynamics and interactions during flooding stress. Plant Physiology 176, 1106–1117. 29097391 10.1104/pp.17.01232 PMC5813540

[CIT0077] Schafer FQ , Buettner GR. 2001. Redox environment of the cell as viewed through the redox state of the glutathione disulfide/glutathione couple. Free Radical Biology & Medicine 30, 1191–1212. 11368918 10.1016/s0891-5849(01)00480-4

[CIT0078] Sjogren CA , Bolaris SC, Larsen PB. 2015. Aluminum-dependent terminal differentiation of the Arabidopsis root tip is mediated through an ATR-, ALT2-, and SOG1-regulated transcriptional response. The Plant Cell 27, 2501–2515. 26320227 10.1105/tpc.15.00172 PMC4815104

[CIT0079] Stahl Y , Simon R. 2005. Plant stem cell niches. International Journal of Developmental Biology 49, 479–489. 10.1387/ijdb.041929ys 16096958

[CIT0080] Sun H , Feng F, Liu J, Zhao Q. 2017. The interaction between auxin and nitric oxide regulates root growth in response to iron deficiency in rice. Frontiers in Plant Science 8, 2169. 29312409 10.3389/fpls.2017.02169 PMC5743679

[CIT0081] Takahashi N , Ogita N, Takahashi T, Taniguchi S, Tanaka M, Seki M, Umeda M. 2019. A regulatory module controlling stress-induced cell cycle arrest in *Arabidopsis*. eLife 8, e43944. 30944065 10.7554/eLife.43944 PMC6449083

[CIT0082] Timilsina R , Kim JH, Nam HG, Woo HR. 2019. Temporal changes in cell division rate and genotoxic stress tolerance in quiescent center cells of Arabidopsis primary root apical meristem. Scientific Reports 9, 3599. 30837647 10.1038/s41598-019-40383-2 PMC6400898

[CIT0083] Van Leene J , Hollunder J, Eeckhout D, et al. 2010. Targeted interactomics reveals a complex core cell cycle machinery in *Arabidopsis thaliana*. Molecular Systems Biology 6, 397. 20706207 10.1038/msb.2010.53 PMC2950081

[CIT0084] Vilarrasa-Blasi J , González-García MP, Frigola D, et al. 2014. Regulation of plant stem cell quiescence by a brassinosteroid signaling module. Developmental Cell 30, 36–47. 24981610 10.1016/j.devcel.2014.05.020

[CIT0085] Wang Z , Rong D, Chen D, Xiao Y, Liu R, Wu S, Yamamuro C. 2021. Salicylic acid promotes quiescent center cell division through ROS accumulation and down-regulation of PLT1, PLT2, and WOX5. Journal of Integrative Plant Biology 63, 583–596. 33017089 10.1111/jipb.13020

[CIT0086] Wein A , Le Gac AL, Laux T. 2020. Stem cell ageing of the root apical meristem of *Arabidopsis thaliana*. Mechanisms of Ageing and Development 190, 111313. 32721407 10.1016/j.mad.2020.111313

[CIT0087] Willemsen V , Bauch M, Bennett T, Campilho A, Wolkenfelt H, Xu J, Haseloff J, Scheres B. 2008. The NAC domain transcription factors FEZ and SOMBRERO control the orientation of cell division plane in Arabidopsis root stem cells. Developmental Cell 15, 913–922. 19081078 10.1016/j.devcel.2008.09.019

[CIT0088] Xu J , Hofhuis H, Heidstra R, Sauer M, Friml J, Scheres B. 2006. A molecular framework for plant regeneration. Science 311, 385–388. 16424342 10.1126/science.1121790

[CIT0089] Yang L , Zhang J, He J, Qin Y, Hua D, Duan Y, Chen Z, Gong Z. 2014. ABA-mediated ROS in mitochondria regulate root meristem activity by controlling PLETHORA expression in Arabidopsis. PLoS Genetics 10, e1004791. 25522358 10.1371/journal.pgen.1004791 PMC4270459

[CIT0090] Yang ZB , Geng X, He C, Zhang F, Wang R, Horst WJ, Ding Z. 2014. TAA1-regulated local auxin biosynthesis in the root-apex transition zone mediates the aluminum-induced inhibition of root growth in Arabidopsis. The Plant Cell 26, 2889–2904. 25052716 10.1105/tpc.114.127993 PMC4145121

[CIT0091] Yi D , Alvim Kamei CL, Cools T, et al. 2014. The Arabidopsis SIAMESE-RELATED cyclin-dependent kinase inhibitors SMR5 and SMR7 regulate the DNA damage checkpoint in response to reactive oxygen species. The Plant Cell 26, 296–309. 24399300 10.1105/tpc.113.118943 PMC3963576

[CIT0092] Yoshiyama KO , Kobayashi J, Ogita N, Ueda M, Kimura S, Maki H, Umeda M. 2013. ATM-mediated phosphorylation of SOG1 is essential for the DNA damage response in Arabidopsis. EMBO Reports 14, 817–822. 23907539 10.1038/embor.2013.112 PMC3790055

[CIT0093] Yu X , Pasternak T, Eiblmeier M, et al. 2013. Plastid-localized glutathione reductase2-regulated glutathione redox status is essential for Arabidopsis root apical meristem maintenance. The Plant Cell 25, 4451–4468. 24249834 10.1105/tpc.113.117028 PMC3875729

[CIT0094] Zhang H , Han W, De Smet I, Talboys P, Loya R, Hassan A, Rong H, Jürgens G, Paul Knox J, Wang MH. 2010. ABA promotes quiescence of the quiescent centre and suppresses stem cell differentiation in the Arabidopsis primary root meristem. The Plant Journal 64, 764–774. 21105924 10.1111/j.1365-313X.2010.04367.x

[CIT0095] Zhang ML , Huang PP, Ji Y, Wang S, Wang SS, Li Z, Guo Y, Ding Z, Wu WH, Wang Y. 2020. KUP9 maintains root meristem activity by regulating K^+^ and auxin homeostasis in response to low K. EMBO Reports 21, e50164. 32250038 10.15252/embr.202050164 PMC7271654

[CIT0096] Zhang W , Swarup R, Bennett M, Schaller GE, Kieber JJ. 2013. Cytokinin induces cell division in the quiescent center of the Arabidopsis root apical meristem. Current Biology 23, 1979–1989. 24120642 10.1016/j.cub.2013.08.008

[CIT0097] Zhang Y , Zheng L, Hong JH, Gong X, Zhou C, Pérez-Pérez JM, Xu J. 2016. TOPOISOMERASE1α acts through two distinct mechanisms to regulate stele and columella stem cell maintenance. Plant Physiology 171, 483–493. 26969721 10.1104/pp.15.01754 PMC4854680

[CIT0098] Zhao Q , Zhou L, Liu J, Cao Z, Du X, Huang F, Pan G, Cheng F. 2018. Involvement of CAT in the detoxification of HT-induced ROS burst in rice anther and its relation to pollen fertility. Plant Cell Reports 37, 741–757. 29464319 10.1007/s00299-018-2264-y

[CIT0099] Zhou W , Lozano-Torres JL, Blilou I, Zhang X, Zhai Q, Smant G, Li C, Scheres B. 2019. A jasmonate signaling network activates root stem cells and promotes regeneration. Cell 177, 942–956. 30955889 10.1016/j.cell.2019.03.006

